# Hepatobiliary scintigraphy may improve radioembolization treatment planning in HCC patients

**DOI:** 10.1186/s13550-016-0248-x

**Published:** 2017-01-05

**Authors:** Manon N. G. J. A. Braat, Hugo W. de Jong, Beatrijs A. Seinstra, Mike V. Scholten, Maurice A. A. J. van den Bosch, Marnix G. E. H. Lam

**Affiliations:** 1 Department of Radiology and Nuclear Medicine, University Medical Center Utrecht, Heidelberglaan 100, 3584 CX Utrecht, The Netherlands; 2 Department of Radiology and Nuclear Medicine, Meander Medical Center, Amersfoort, The Netherlands

**Keywords:** Hepatobiliary scintigraphy, Radioembolization, Hepatotoxicity, Yttrium-90

## Abstract

**Background:**

Routine work-up for transarterial radioembolization, based on clinical and laboratory parameters, sometimes fails, resulting in severe hepatotoxicity in up to 5% of patients. Quantitative assessment of the pretreatment liver function and its segmental distribution, using hepatobiliary scintigraphy may improve patient selection and treatment planning. A case series will be presented to illustrate the potential of this technique.

Hepatocellular carcinoma patients with cirrhosis (Child-Pugh A and B) underwent hepatobiliary scintigraphy pre- and 3 months post-radioembolization as part of a prospective study protocol, which was prematurely terminated because of limited accrual. Included patients were analysed together with their clinical, laboratory and treatment data.

**Results:**

Pretreatment-corrected ^99m^Tc-mebrofenin liver uptake rates were marginal (1.8–3.0%/min/m^2^), despite acceptable clinical and laboratory parameters. Posttreatment liver functions seriously declined (corrected ^99m^Tc-mebrofenin liver uptake rates: 0.6–2.4%/min/m^2^), resulting in lethal radioembolization-induced liver disease in two out of three patients.

**Conclusions:**

Hepatobiliary scintigraphy may be of added value during work-up for radioembolization, to estimate liver function reserve and its segmental distribution, especially in patients with underlying cirrhosis, for whom analysis of clinical and laboratory parameters may not be sufficient.

## Background

Transarterial radioembolization (RE) is an emerging treatment option for patients with hepatocellular carcinoma (HCC). Comparative studies have shown that RE outperforms transarterial chemoembolization (TACE) with regard to overall survival and time to progression, with a similar toxicity profile [1, 2]. Similar results were reported in a recent randomised controlled trial in patients with HCC Barcelona Clinic Liver Cancer stage A/B (the Premiere trial; NCT00956930 [3]). Treatment planning, however, is a balancing act between optimal efficacy and acceptable toxicity.

Lodging of radioactive microspheres in the functional liver parenchyma may result in radiation damage, consistent with sinusoidal obstruction syndrome at histopathology [4]. The extent of the radiation damage depends on the percentage of the functional liver within the treated volume, the tumour-to-non-tumour ratio (TNR) and the regenerative capacity of the remaining functional liver parenchyma.

The functional liver remnant after RE is hard to predict, due to the heterogeneity of the radiation-absorbed dose distribution, but may be crucial to prevent (severe) radioembolization-induced liver disease (REILD), especially in patients with a marginal liver function, as seen in cirrhosis or after chemotherapy.

Assessment of eligibility for RE is usually based on a combination of clinical, laboratory and imaging parameters, with special attention to performance score, bilirubin, albumin, portal vein thrombosis and ascites. Nonetheless, this evaluation can sometimes not predict serious toxicity after RE, with incidences of lethal REILD up to 5% in large series [5]. There is definitively room for improvement.

Hepatobiliary scintigraphy with technetium-99m (^99m^Tc)-mebrofenin is a dynamic quantitative liver function test; for example able to assess severity of fibrosis in hepatitis C-positive patients [6]. Furthermore, it can adequately predict the risk of postoperative liver failure, outperforming the Child-Pugh score and CT volumetry [7–9]. De Graaf et al. reported that a liver remnant function cut-off value of 2.69%/min/m^2^ (=body surface area corrected ^99m^Tc-mebrofenin hepatic uptake rate = cMUR) can accurately identify patients at risk for postoperative liver failure, regardless of the presence of underlying liver disease [7]. Moreover, an uptake below 2.2%/min/m^2^ was reported to be associated with a 50% risk of lethal postoperative liver failure [8].

We hypothesised that quantitative assessment of remnant liver function using hepatobiliary scintigraphy can improve patient selection, complementary to routine assessment. In this case series, three cases with pre- and posttreatment hepatobiliary scintigraphies will be presented to illustrate the potential of this technique.

## Methods

### Patient selection

We reviewed the data of patients, who were initially included in a multicenter randomised controlled trial in 2012 comparing TACE and RE in patients with unresectable HCC (the TRACE study; NCT01381211 [10]). Unfortunately, this trial was prematurely terminated due to the lack of inclusions. Ultimately, in our hospital, three patients were included before the study closure. The medical ethics committee of our institution waived the need for informed consent for review of the imaging data.

### Treatment

Before treatment, all patients underwent screening with dynamic contrast-enhanced magnetic resonance imaging (MRI), bone scintigraphy and angiography. Subsequently, a surrogate particle, ^99m^Tc-macroaggregated albumin (^99m^Tc-MAA) (TechneScan LyoMaa, Mallinckrodt Medical, Petten, The Netherlands) was intra-arterially injected, directly followed by a ^99m^Tc-MAA planar scintigraphy and single-photon emission computed tomography (SPECT)/CT. The ^99m^Tc-MAA SPECT/CT was used to calculate the lung shunt fraction (LSF) and to detect other extrahepatic deposition.

RE was performed using yttrium-90 (^90^Y)-labelled glass microspheres (Theraspheres®, BTG International, London, England) according to international guidelines [11]. On the same day, a ^90^Y-positron emission tomography (PET)/CT (mCT, Siemens Healthcare, Erlangen, Germany) was performed to assess the activity distribution. Our acquisition protocol was published earlier [12].

### Hepatobiliary scintigraphy

Additional to the standard work-up, patients underwent a ^99m^Tc-mebrofenin hepatobiliary scintigraphy prior to and 3 months after RE. Our acquisition protocol is triphasic, similar to the protocol previously described by de Graaf et al. [13]. A dual-head gamma camera (Symbia 16T, Siemens Healthcare, Erlangen, Germany) is positioned over the patient, including the heart and liver in the field of view. After intravenous administration of 200 MBq ^99m^Tc-mebrofenin (Bridatec, GE Healthcare), 36 dynamic anterior and posterior planar images are acquired over 10 min at 10 s per frame, using a low-energy high-resolution collimator (matrix 128 × 128; energy window 130–150 keV). Subsequently, a fast SPECT/CT is performed (matrix 128 × 128; 64 projections in total at 8 s/projection). A low dose CT is acquired for attenuation correction and anatomical reference. Thereafter, a second series of planar scintigraphic images is performed to evaluate biliary secretion (matrix 128 × 128; 30 frames at 60 s/frame).

### Image analyses

Analysis of the hepatobiliary scintigraphies was not performed until after treatment (in 2016).

The hepatobiliary scintigraphy data were processed using in-house developed software (Volumetool [14]), similar to the method described by de Graaf et al. [13]. A geometric mean dataset was calculated from the anterior and posterior planar projections of the first dynamic series (G_mean_ = √(anterior × posterior)). Regions of interest (ROI’s) were drawn on the planar G_mean_ dataset around the total image, cardiac blood pool and whole liver to calculate the ^99m^Tc-mebrofenin liver uptake rate (expressed in %/min), as previously described by Ekman et al. [15]. This value was divided by the body surface area (BSA) to correct for inter-patient variability in metabolic needs (cMUR, expressed as %/min/m^2^). Liver and heart ROI's were placed so as to avoid spillover from one to the other. Subsequently, the whole liver was manually delineated on the SPECT/CT images, as well as the treated and non-treated volumes after correlation with posttreatment ^90^Y-PET/CT, enabling assessment of both the volumes and contribution to the liver function. The latter was done by dividing the sum of counts in the ROI of the treated volume by the total liver counts, representing the contribution of the treated volume to the total cMUR (as calculated on the G_mean_ dataset). The contribution of the non-treated volume was identically assessed. Activity in the hilar and extrahepatic bile ducts was excluded from the ROI’s to avoid falsely increased regional activity due to biliary excretion.

The tumour-absorbed dose and non-tumorous liver-absorbed dose of the treated liver parenchyma were calculated using ROVER software (ABX-CRO Advanced Pharmaceutical Services, Dresden, Germany). For each patient, a volume of interest (VOI) was drawn on the ^90^Y-PET/CT in the tumours and in the non-tumorous treated liver tissue. To prevent erroneous overlap, the pretreatment CT was used as a reference. The mean activity in becquerel in the VOI was computed. Subsequently, a correction factor was applied to correct for the low number of positrons per becquerel. The corrected activity at the time of ^90^Y-PET acquisition was recalculated to the corrected activity at time of treatment by adjustment for the radioactive decay. Subsequently, the healthy liver absorbed doses were calculated as follows: Healthy liver absorbed dose (Gy) = ((50 Gy * Kg/GBq) x (corrected activity in GBq)) / (VOI volume (mL) * 1.06 g/mL/1000).

## Results

### Case 1

A 74-year-old male with a history of liver cirrhosis due to alcohol abuse was diagnosed with a mass in the right liver lobe at ultrasonography. A subsequent liver CT revealed a multifocal, hypervascular mass in segments 5 and 8 of the liver with contrast washout, consistent with HCC. The largest tumour measured 3.7 cm (tumour involvement 1%). Cirrhosis, splenomegaly and gastro-esophageal varices were also present, but no ascites or portal vein thrombosis. The day of treatment, he was graded as Child-Pugh grade A6/ALBI score grade 1 (Table 1).

**Table 1:** Liver biochemistry tests during follow up

Case	Time to RE (days)	Bilirubin (mg/dL)	Albumin (g/L)	AST (U/L)	ALT (U/L)	ALP (U/L)	INR	Ascites	AFP (μg/L)
1	−1	0.9	31	72	60	252	1.24^a^	no	750
	+12	1.2	30	75	51	326	–	minimal	–
	+35	1.1	27	107	59	290	3.49^a^	–	–
	+65	1.1	24	60	47	286	5.79^a^	diffuse	–
	+86	2.4	20	62	36	347	1.36^a^	–	–
2	−1	1.9	27	76	60	115	1.38	minimal	140
	+14	4.3	25	96	58	138	–	moderate	–
	+18	6.7	22	72	84	67	1.8	diffuse	–
	+33	5.6	25	69	45	100	0.9	diffuse	–
	+61	8.2	23	69	170	126	1.7	massive	–
	+95	11.1	23	250	212	153	–	massive	36
3	0	1.6	25	61	46	122	1.18	minimal	270
	+14	2.5	28	80	50	143	1.16	moderate	210
	+30	2.3	22	58	32	126	1.20	moderate	–
	+71	2.2	23	63	37	144	1.20	moderate	–
	+160	2.9	23	67	32	156	1.20	massive	5200

*AFP* alpha-fetoprotein, *ALP* alkaline phosphatase, *ALT* alanine aminotransferase, *AST* aspartate aminotransferase, *INR* international normalized ratio

^a^ = under treatment with coumarin derivates

The LSF was 9%. He underwent a right lobar treatment (3,1 GBq, target dose 80 Gy). Posttreatment ^90^Y-PET/CT showed adequate targeting of the lesion in segments 5 (306 Gy) and 8 (376 Gy). The average absorbed dose of the non-tumorous liver parenchyma was 33 Gy.

Twelve days after treatment, he visited the outpatient clinic with complaints of increasing abdominal girth and weight. He was treated with a low sodium diet and diuretics. Six weeks thereafter, he was readmitted because of decompensated cirrhosis with worsening encephalopathy. At 3-month follow-up, laboratory tests showed grade 2 bilirubin (2.4 mg/dL), grade 3 albumin (20 g/L), grade 1 ALP, AST and ALT toxicity and an elevated ammonia serum value (60 μmol/L). Concurrent liver CT showed ascites in all quadrants, shrinkage of the liver and partial necrosis of the HCC’s (partial response of the smaller tumours and stable disease of the largest tumour).

Evaluation of the hepatobiliary scintigraphies showed a whole liver function decline from 3.0 to 2.4%/min/m^2^ (Table 2). The function of the treated right hemiliver declined from 2.3 to 1.6%/min/m^2^, without evident hypertrophy of the left hemiliver (0.8 vs 0.7%/min/m^2^).

**Table 2:** Hepatobiliary scintigraphy measurements at baseline and 3-month follow-up

		Baseline			3-month follow-up		
Case		Liver (total)	Liver (treated)	Liver (non-treated)	Liver (total)	Liver (treated)	Liver (non-treated)
1	Volume	2481	1845	636	1919	1357	562
	%	100	74	26	100	71	29
	cMUR	3.0	2.3	0.7	2.4	1.6	0.8
	%	100	77	23	100	67	33
2	Volume	1396	1178	285	1111	855	256
	%	100	84	16	100	77	23
	cMUR	1.8	1.6	0.2	0.6	0.4	0.2
	%	100	87	13	100	77	23
3	Volume	1288	881	407	1218	713	505
	%	100	68	32	100	59	41
	cMUR	2.2	1.5	0.7	1.8	0.8	1.0
	%	100	70	30	100	43	57

Volume (in mL); cMUR = BSA corrected ^99m^Tc-mebrofenin uptake rate (in %/min/m^2^)

He died 4 months after RE due to hepatic failure, probably caused by REILD.

### Case 2

A homeless 57-year-old male with a history of cirrhosis due to alcohol abuse was diagnosed with a multifocal HCC. MRI liver revealed four hypervascular lesions in segments 1 (1.0 cm), 5 (4.4 cm) and 8 (3.2 and 0.8 cm), consistent with HCC (tumour involvement 1%). Coexisting liver cirrhosis, portal hypertension and a moderate amount of ascites was also present, but no portal vein thrombus. At diagnosis, he had decompensated cirrhosis, which was recompensated 5 months later at treatment. He had a Child-Pugh grade B8 at treatment (ALBI grade 3) (Table 1). The LSF was 4%.

**Fig. 1:**
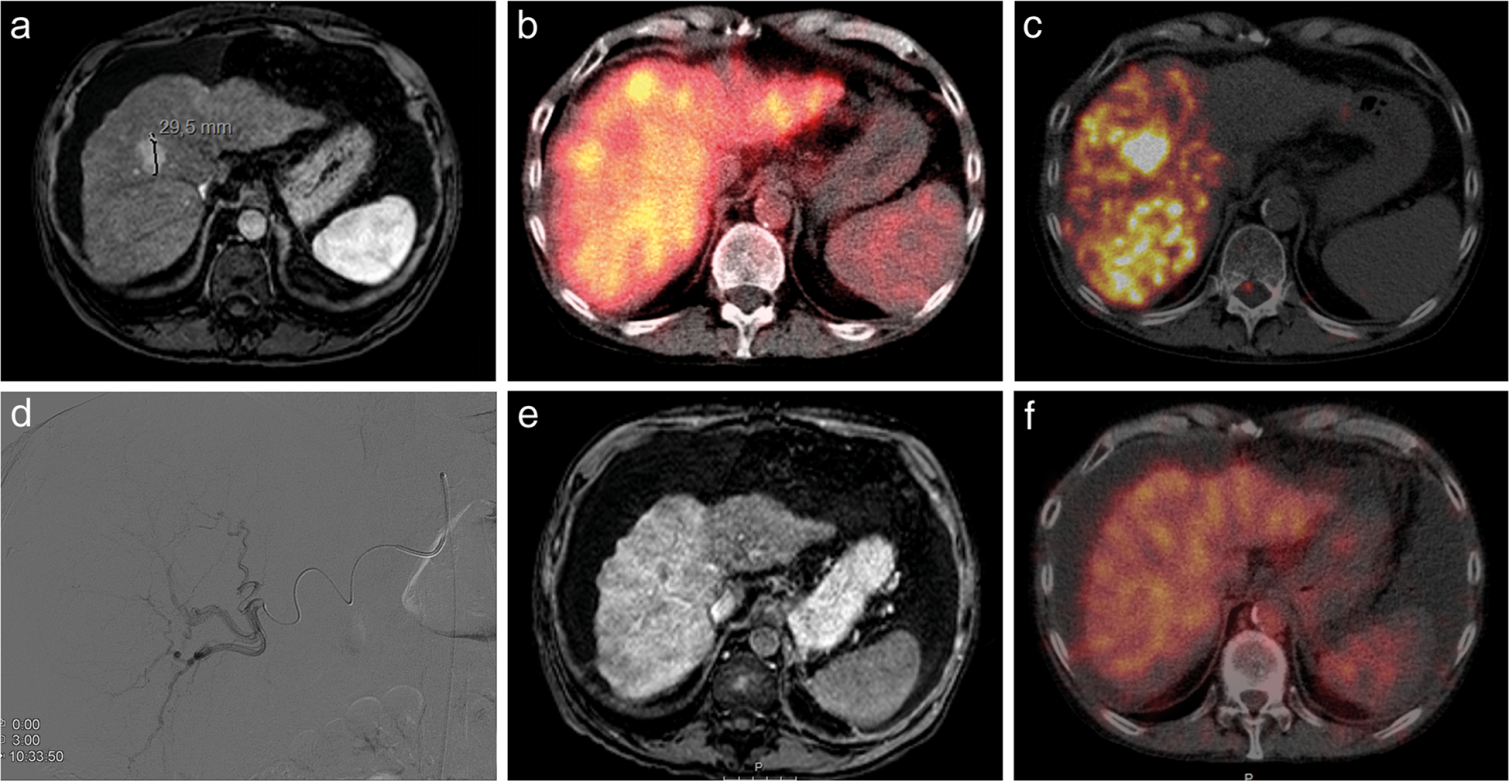
Pre- and posttreatment images of case 2. On the arterial phase of the pretreatment MRI, a hypervascular lesion in segment 8 is depicted on a background of liver cirrhosis, consistent with a HCC (**a**). Pretreatment hepatobiliary scintigraphy shows a visually fairly homogenous ^99m^Tc-mebrofenin uptake with a defect in segment 8, corresponding to the HCC (**b**). Posttreatment ^90^Y-PET/CT shows good targeting of the HCC (227 Gy), but also a significant ^90^Y deposition in segment 7 (91 Gy) (**c**). At treatment angiography, the endhole catheter tip was positioned at the bifurcation of the anterior and posterior right hepatic artery. The overdosage of the posterior sector is therefore most likely due to preferential flow (**d**). Posttreatment MRI and hepatobiliary scintigraphy show ascites and shrinkage of the cirrhotic liver with increased arterial enhancement of the treated lobe and decreased ^99m^Tc-mebrofenin uptake (**e**, **f**)

He underwent a right lobar treatment (2.5 GBq, target dose 100 Gy). The posttreatment ^90^Y-PET/CT showed reasonable targeting, but also a relatively large amount of activity in the tumour-free segments 6 and 7 with an average absorbed dose of 91 Gy (Fig. 1). The absorbed dose for the tumours in segments 1, 5 and 8 was 226, 63 and 227 Gy, respectively. The absorbed dose of the smallest tumour (0.8 cm) could not reliably be measured.

Fourteen days after treatment, he was readmitted with increasing ascites and peripheral oedema, consistent with decompensated cirrhosis. Two days later, he developed a spontaneous bacterial peritonitis, successfully managed with albumin suppletion and antibiotics.

At 3-month follow-up, his liver function had further declined to Child Pugh grade C11, with a grade 3 bilirubin toxicity (Table 1). Follow-up MRI at that time showed a partial response of all four lesions, consistent with the decrease in AFP levels. However, also massive ascites and shrinkage of the liver were noted.

At 3-month follow-up, his liver function had declined to 0.6%/min/m^2^ (1.8%/min/m^2^ at baseline). The pre-RE liver function was mainly located in the right hemiliver (1.6%/min/m^2^). After RE, this declined to 0.4%/min/m^2^, without compensatory function increase of the left hemiliver (stable at 0.2%/min/m^2^).

**Fig. 2:**
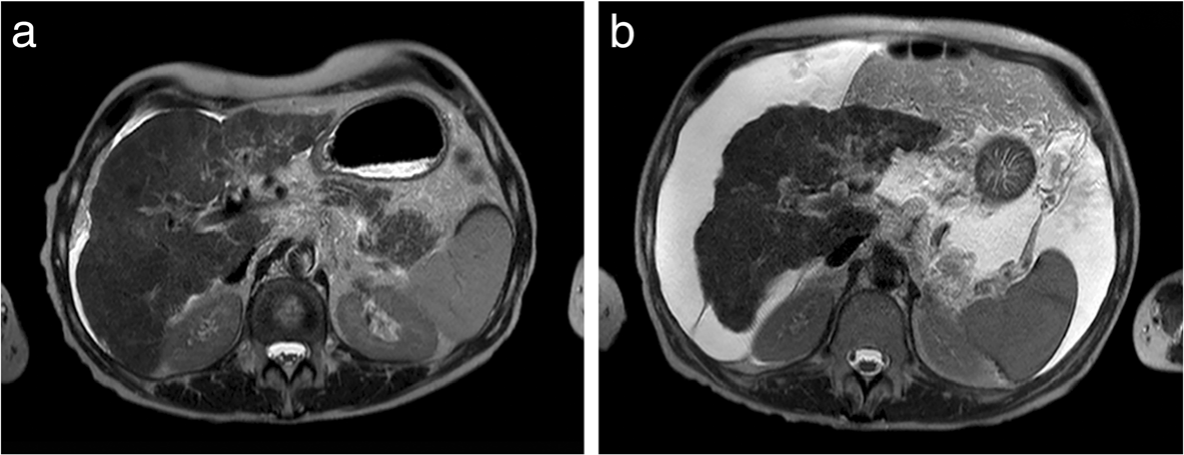
Pre- and posttreatment T2-weighted images of case 2. On the pretreatment image (**a**), a nodular surface of the liver is seen, consistent with cirrhosis. A small amount of ascites is present. Three months after treatment (**b**), the liver has atrophied impressively and the amount of ascites has substantially increased, consistent with REILD

He died 4 months after RE treatment due to definite REILD (Fig. 2).

### Case 3

A 74-year-old male with a history of hepatitis B and cirrhosis (Child Pugh grade C11/ALBI grade 3) was diagnosed with multifocal HCC, which was initially left untreated because of decompensated cirrhosis. After 6 months, he was admitted because of severe haemorrhage of one lesion. He was treated with bland coiling of the arterial supply to this lesion. Another 12 months later, his cirrhosis had recompensated (Child Pugh grade B8/ALBI grade 2) and he opted for RE. At that time, his MRI revealed a large HCC located in segment 7/8 (6.6 cm), in segment 4 (6.3 cm) and two smaller lesions in segment 6 (tumour involvement 19%). No portal vein thrombus was present. The LSF was 11%.

He underwent a right lobar treatment (2 GBq; target dose 120 Gy). The posttreatment ^90^Y-PET/CT showed adequate targeting of lesions in segment 6 (243 and 422 Gy) and segment 7/8 (309 Gy). The average absorbed dose of the non-tumorous parenchyma was 32 Gy. Subsequent treatment of segment 4 was cancelled, because of uncorrectable extrahepatic deposition and a LSF of 25% on ^99m^Tc-MAA SPECT/CT 1 month later.

**Fig. 3:**
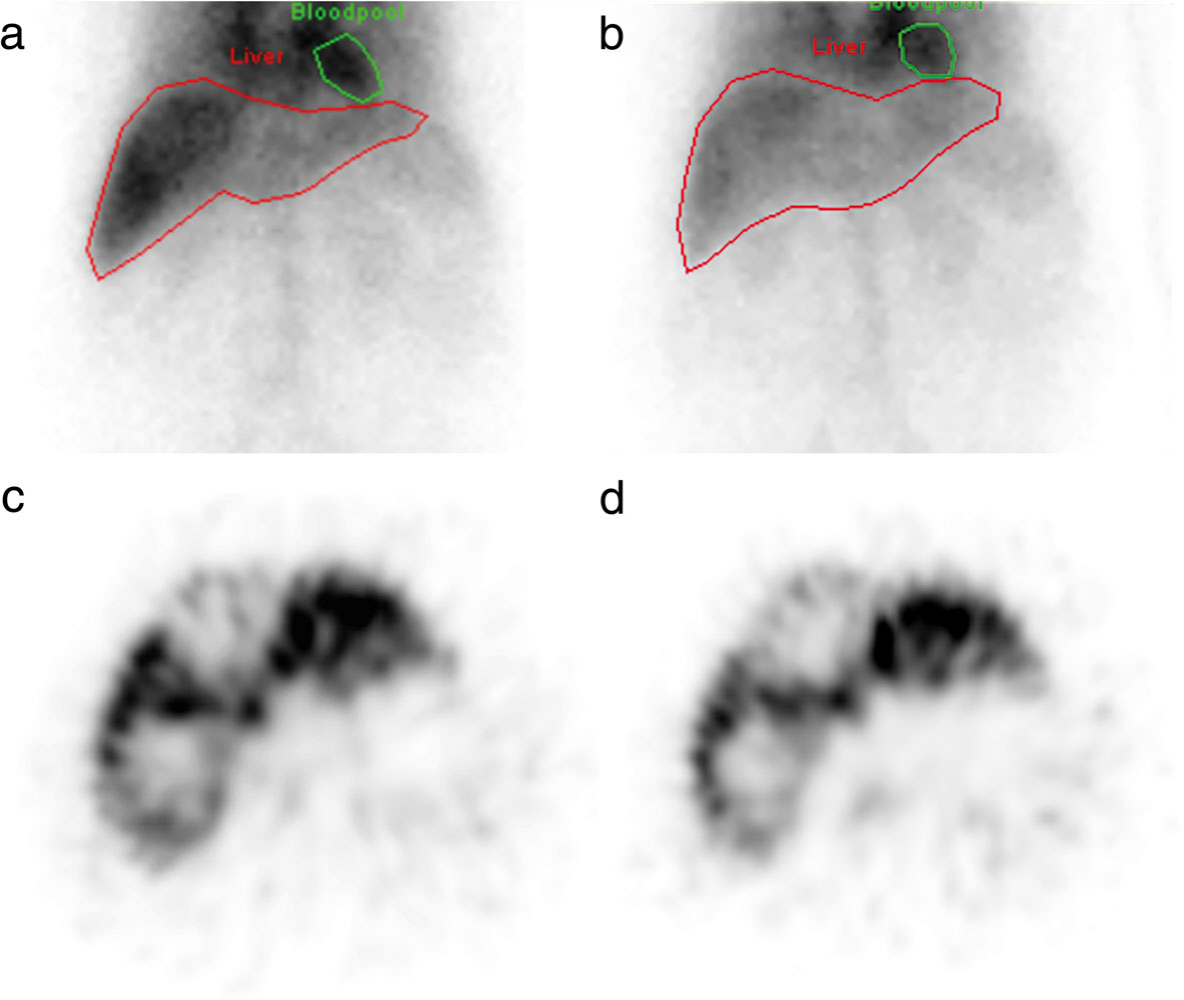
Pre- and posttreatment hepatobiliary scintigraphy images of case 3. The pretreatment G_mean_ planar image (**a**) shows that ^99m^Tc-mebrofenin is mainly taken up in the right hemiliver. Centrally, in the liver (segment 4), no uptake is seen, due to the presence of a large HCC (**c**). After treatment (G_mean_ planar (**b**), SPECT (**d**)), a decrease in total liver uptake is seen, primarily due to a decrease in uptake in the right hemiliver. The left hemiliver has hypertrophied slightly (**b**: *red line*, contouring the functional liver). On the SPECT images (**c**, **d**), the uptake in the left hemiliver is similar to the pretreatment image. The defect in the uptake in segment 4 remains, consistent with untreated segment 4 lesion (**d**)

Follow-up MRI at 3 and 6 months showed a partial response of the lesions in segments 6 and 7/8, but progressive growth of the lesion in segment 4. At 3-month follow-up, the function of the treated liver volume had declined to 0.8%/min/m^2^ (1.5%/min/m^2^ at baseline), whereas the non-treated volume showed a minimal functional improvement from 0.7 to 1.0%/min/m^2^ (Fig. 3), resulting in a decline of the total liver function from 2.2 to 1.8%/min/m^2^.

He died 1 year after RE due to tumour progression (not due to REILD).

## Discussion

In this case series, we presented three cases with a dismal outcome after lobar ^90^Y glass microspheres RE treatment. The rapid clinical deterioration in the first two patients was due to REILD. In all three patients, pretreatment clinical, laboratory and imaging parameters were within acceptable limits for safe treatment, but did not predict the severe toxicity encountered, even though all cases involved lobar treatments only.

Patients amendable for RE commonly have a compromised liver function due to cirrhosis or other underlying liver disease (e.g. prior chemotherapy). Most large published series applied limited exclusion criteria regarding the liver function, often confined to a total bilirubin of <2 or <3 mg/dL [2, 16]. Consequently, 18–44% of patients, who underwent RE for HCC, had a Child-Pugh B cirrhosis and in up to 2% even a Child-Pugh C cirrhosis [2, 16]. In patients who undergo segmental or lobar treatment only, this is generally accepted. However, contrary to hepatectomy candidates, pretreatment quantitative segmental assessment of the liver function is often lacking [17]. In other words, is the liver function of the non-treated lobe sufficient to compensate for radiation damage in the treated part of the liver?

Adequate assessment of the overall liver function is difficult due to the diversity of its functions (e.g. detoxification, synthesis). Several clinical scoring systems (incorporating some of these functions) exist to estimate overall survival and eligibility for hepatectomy in patients with cirrhosis [18]. Of these, the Child-Pugh score is best-known. However, considerable heterogeneity exists within the Child-Pugh categories, resulting in an unreliable prediction of liver failure after hepatectomy [19, 20]. The recently introduced ALBI scoring system might be more successful in these predictions [19]. The ALBI score has shown to be a successful predictor of survival after RE, though its correlation with hepatotoxicity is not known [21].

Contrary to the clinical scoring systems, hepatobiliary scintigraphy with SPECT/CT allows for quantification of a non-uniform distribution of the liver function, as often seen in cirrhosis [13]. The visualisation and quantification of possible regional differences in liver function can be crucial in large liver resections or segmental liver-directed treatments, as illustrated by our cases. In all cases, a right lobar treatment was performed to spare the non-tumorous liver parenchyma. Unfortunately, most of the functional liver tissue was located in the treated right hemiliver (70–87%), resulting in considerable liver function decline, both clinically and at hepatobiliary scintigraphy. Prior knowledge of these segmental differences probably would have led to a change in treatment strategy or renouncement [17, 22].

De Graaf et al. suggested a cMUR cut-off value of 2.69%/min/m^2^, after analysis of hepatobiliary scintigraphies of 55 patients before and after major hepatectomy [7]. Nine of their patients developed postoperative liver failure (8/9 with compromised livers), which was lethal in 8/9 cases. The cut-off value of 2.69%/min/m^2^ rightly identified all but one patient with postoperative liver failure. In contrary, CT volumetry based cut-off values failed to identify two cases and in case of BSA corrected volumes even three cases. Furthermore, for hepatobiliary scintigraphy, one cut-off value sufficed for both compromised and normal livers, whereas cut-off values for CT volumetry varied.

The cut-off value of 2.69%/min/m^2^ implies a serious risk of liver failure in the presented cases, of whom two developed fatal REILD. Interestingly, the data in Table 2 show no compensatory hypertrophy of the non-treated lobes in the first two cases, indicative of a severely compromised liver regeneration. However, in the third case, the non-treated lobe had hypertrophied (25% volume increase) with a liver function increase of 43% from 0.7 to 1.0%/min/m^2^. The regeneration capacity in this case may explain the longer survival, as hypertrophy is known to continue up to 12 months after RE [23].

According to the device instruction manual for glass microspheres [24], a target dose of 120 Gy (range 80–150 Gy) is suggested for the treated liver volume, in principle ignoring the presence of underlying liver disease, TNR and liver volume (total volume and % treated volume). Yet, a definite relationship exists between the absorbed dose in the non-tumorous parenchyma (D_ntp_) and hepatotoxicity [25, 26]. Chiesa et al. [25] identified a tolerance dose of 75 Gy for 15% RE-related hepatotoxicity (=TD_15 whole liver_) in 43 HCC patients with a Child-Pugh A cirrhosis after lobar RE with glass microspheres (based on the ^99m^Tc-MAA SPECT data). In contrary, 83% of their Child-Pugh B7 patients had RE-related liver failure at a mean liver dose <60 Gy [27]. All Child-Pugh A patients (6/43) with hepatotoxicity had a mean liver dose >60 Gy and at least 58% of the liver volume treated. Our findings are consistent with these results.

Logically, also a positive relationship exists between tumour dose (D_tumour_) and tumour response [25, 26, 28]. Garin et al. [29] suggested a ^99m^Tc-MAA SPECT/CT based tumour response threshold dose (TTD) of 205 Gy for HCC treatment with glass microspheres, using measurements similar to ours (2 cc VOI in tumour and non-tumorous tissue). This TTD is consistent with the findings of Chiesa et al. (217 Gy) [25]. However, larger lesions require a higher D_tumour_; i.e. >1000 Gy for lesions >10 cc to achieve 50% tumour control probability [25]. In our patients, three out of four lesions >10 cc were treated with >217 Gy (case 1: 306 Gy/case 2: 63 and 227 Gy/case 3: 309 Gy). Yet, all lesions showed partial response, except the lesion in case 1. Nonetheless, target dose and volume planning in case of larger and/or necrotic lesions is more difficult. In these cases, extra caution has to be taken with regard to treatment intensification or retreatments to prevent serious hepatotoxicity, especially in the presence of cirrhosis. Hepatobiliary scintigraphy may help in this patient selection.

This case series is obviously limited by patient number, caused by preliminary closure of the TRACE study in our hospital. However, it is the first case series on liver-directed RE that shows the potential benefit of imaging-based quantification of liver function and its segmental distribution, complementary to systemic assessment of overall liver function. Patients with a marginal pretreatment liver function, as suspected after routine evaluation, may be further screened by hepatobiliary scintigraphy for improved treatment planning. Definition and validation of thresholds for safe treatment should be based on clinical data. Large studies should answer questions with regard to the minimal acceptable remaining liver function and the numeric relation between the absorbed dose to the functional liver parenchyma and the decline in liver function posttreatment.

## Conclusions

Based on this case series, hepatobiliary scintigraphy seems to be complementary to current patient selection based on clinical, laboratory and imaging parameters alone. Although thresholds for safe treatment should be determined and validated in large patient series, the potential of hepatobiliary scintigraphy to quantify segmental liver function, in contrast to overall liver function, seems imperative for RE treatment planning.
